# Deep Intronic SVA_E Retrotransposition as a Novel Factor in Canavan Disease Pathogenesis

**DOI:** 10.1089/hum.2025.006

**Published:** 2025-04-21

**Authors:** Melina Weiß, Mareike Selig, Johannes Friedrich, Anna Wierczeiko, Stefan Diederich, Helen Sigel, Janna Bredow, Florian S. Eichler, Amanda Nagy, Denise Seyler, Laura Holthöfer, Susanne Gerber, Susann Schweiger, Matthias Linke, Annette Bley

**Affiliations:** 1 Institute for Human Genetics, University Medical Center of the Johannes Gutenberg University Mainz, Germany; 2 University Medical Center Hamburg-Eppendorf, University Children’s Hospital, Hamburg, Germany; 3 Department of Neurology, Massachusetts General Hospital, Boston, Massachusetts, USA; 4 Leibniz Institute for Resilience Research, Mainz, Germany; 5 Institute of Molecular Biology (IMB), Mainz, Germany.

**Keywords:** Canavan disease, retrotransposon, long-read sequencing

## Abstract

Canavan disease (CD) is a rare autosomal recessive leukodystrophy caused by biallelic pathogenic variants in the *ASPA* gene. CD is characterized by developmental delay, macrocephaly, and abnormal muscle tone. The biochemical diagnosis is confirmed by increased *N*-acetylaspartic acid levels. The phenotypic presentation varies, with 85–90% of individuals exhibiting the severe, typical form, while 10–15% present with a milder, atypical form. Here we report on five patients with a clinical and biochemically proven diagnosis in whom a second pathogenic variant had not yet been identified. Targeted long-read sequencing of the entire *ASPA* gene revealed an SVA_E retrotransposable element located in intron 4 that had been missed by standard short-read-based diagnostic procedures. Haplotype analysis of all patients showed linkage of the SVA_E element with a noncoding variant in intron 1. Functional characterization of the SVA_E element suggests that transcripts of the affected allele are prone to highly efficient mRNA degradation processes. These findings enhance the precision of genetic diagnostics and enable improved guidance for families as well as facilitating potential access to targeted therapies.

## INTRODUCTION

Canavan disease (CD, OMIM # 271900) is an autosomal recessive inherited metabolic disease caused by biallelic pathogenic variants in *ASPA* that lead to a deficiency of the enzyme aspartoacylase (ASPA) (OMIM * 608034). The loss of ASPA activity results in an accumulation of *N*-acetylaspartic acid (NAA) in the brain and other parts of the body.^1^ NAA is thought to function as a molecular water pump causing spongiform degeneration of the brain white matter and leading to fatal brain disease.^2^ The exact prevalence of CD is unknown. It is reported to be particularly high in the Ashkenazi Jewish population.^3^

Patients with the classical phenotype of CD present with hypotonia, macrocephaly, visual impairment, feeding difficulties, and seizures. Neurodevelopmental delay usually becomes evident in the first year of life and is frequently followed by developmental regression later in childhood or adolescence. For most individuals with CD, life expectancy is reduced.^4^ The phenotypic presentation varies, with 85–90% of individuals exhibiting the severe, typical form, while 10–15% present with a milder, atypical form, reaching advanced psychomotor milestones. This phenotypic spectrum might correlate with residual ASPA enzyme activity and urine NAA levels.^5,6^ If clinically suspected, the diagnosis of CD can usually be confirmed by pathological elevation of NAA in urine, blood, or MR spectroscopy (MRS), and/or by the identification of pathogenic variants in the *ASPA* gene through molecular genetic analysis.^7^ The *ASPA* gene is located on chromosome 17p13.2, encoding for six exons and spanning ~30 kb of DNA.^8^ Currently there is no approved curative treatment available but gene therapy trials are ongoing (NCT04833907, NCT04998396). Sufficient counseling of the families, including prenatal and preimplantation diagnostics and gene-modifying therapy, is only possible if both pathogenic variants are known.

In our study cohort of more than 55 CD patients, ~13% of patients remained incompletely diagnosed as no or only one pathogenic variant in the *ASPA* gene had been detected.

Retrotransposons are mobile genetic elements that use reverse transcription to move within the genome. They are classified into two main categories: long terminal repeat (LTR) retrotransposons and non-LTR retrotransposons, which include long interspersed nuclear elements (LINEs) and short interspersed nuclear elements (SINEs).^9,10^ Among SINEs, SVA elements are composite structures incorporating SINE, variable number of highly GC-rich tandem repeats (VNTR), and Alu sequences. These evolutionary young elements are mobilized by the LINE-1 reverse transcriptase and can impact the genomic integrity through mechanisms such as insertional mutagenesis, exon shuffling, aberrant splicing, and the creation of differentially methylated regions.^11^ While SVA elements contribute to genetic diversity and evolution,^12^ they are also associated with disease pathogenesis.^13,14^

Recently, long-read sequencing (LRS) approaches contributed to a complete sequence of the human genome (T2T-CHM13v2.0) overcoming the highly fragmented and unresolved portions of previous human genome assemblies^15,16^ and are increasingly used in clinical settings.^17,18^ As a major LRS technology, nanopore-based sequencing has made continuous advances in chemistry and algorithms, leading to improved sequencing accuracy in single-nucleotide variants (SNVs) and indel calling.

Here we used a modified gene-specific target enrichment using CRISPR/Cas9 followed by LRS^19,20^ to comprehensively analyze the gene structure of *ASPA* in five patients in our cohort presenting with a clinical and biochemical diagnosis of CD who were lacking a second pathogenic *in trans* variant. Furthermore, the impact of the newly identified intronic *ASPA* variants on mRNA structure was studied by using LRS.

## MATERIALS AND METHODS

### Participants

Skin biopsies of five individuals (patients I–V) with clinical symptoms and biochemically proven diagnosis of CD (Table 1) were collected. Skin biopsies were used to culture fibroblasts. Clinical information was assessed within the PeriNAA natural history study, University Children’s Hospital, Hamburg, Germany. MR images (MRI) were available for patients I, II, III, and V. MRS was done in patients I and II (Supplementary Fig. S1).

Informed consent was obtained from all subjects involved in the study. The study was conducted in accordance with the Declaration of Helsinki, and for patients I–V approved by the Ethics Committee of the Medical Association of Hamburg (Germany) for studies involving humans (PV3782).

### Cell culture and DNA/RNA isolation

The cells were cultured under sterile conditions in Iscove’s modified Dulbecco’s medium, with 10% fetal calf serum and 1% penicillin–streptomycin until passage 9. Genomic DNA was extracted from the fibroblasts of all patients using the Monarch high molecular weight (HMW) DNA Extraction Kit (NEB) and total RNA was isolated using the High Pure RNA Isolation Kit (Roche) according to the manufacturer’s instructions.

### Short-read whole-genome sequencing

The Illumina KAPA HyperPrep Kit (Roche) and the NovaSeq6000 sequencing platform (Illumina) were employed for library preparation and high-throughput sequencing of patient III as part of an extended routine diagnostics.

### Targeted DNA nanopore sequencing (LRS, Oxford Nanopore Technologies, ONT)

CRISPR/Cas9 enrichment of the *ASPA* gene was modified followed by LRS.^19,20^ CRISPR/Cas9 crRNA sequences were designed using the CHOPCHOP tool^21^ and ordered from Integrated DNA Technologies (IDT). Briefly, 5 μg HMW-DNA was used as input. The 3′-OH ends were blocked by simultaneous addition of 0.1 mM ddGTP, 0.25 mM CoCl_2_, 20 U terminal transferase, and 10× Tdt buffer. The reaction mixture was incubated for 2 h at 37°C, followed by 20 min at 75°C. Then 5′-phosphate residues were removed by dephosphorylation, and new DNA ends were created by Cas9-gRNAs targeting the *ASPA-*specific sequence (Supplementary Table S1). Cas9 was digested with Proteinase K by incubation for 10 min at 65°C and 15 min at 70°C to inactivate Proteinase K. After enzymatic digestion of Cas9 and dA-tailing, sequencing adapters were ligated to the cleavage sites for subsequent library preparation (ONT, SQK-LSK114) and sequenced on single R10.4.1 flow cells (ONT, FLO-PRO114M).

### cDNA nanopore sequencing of patient III

The integrity of the samples’ total RNA was assessed using the RNA 6000 Nano Kit (Agilent), and 1.5 μg of RNA with an RNA integrity number of above 8.3 was used for library preparation of patient III with the cDNA-PCR Sequencing Kit V14 (ONT, SQK-PCS114). In total, 2.5 μg of were was sequenced on a single R10.4.1 flow cell (ONT, FLO-PRO114M). The protocol is optimized for identification and quantification of full-length transcripts with high output.

### Reverse transcription-PCR followed by LRS of patients I, II, IV, and V

First-strand cDNA was synthesized using the Prime-Script RT MasterMix Kit (TaKaRa Bio) with 500 ng of RNA (patients I, II, IV, and V). The reverse transcription (RT)-PCR assay is based on an exon-bridging approach (Supplementary Table S1) and includes exons 5 and 6 of the *ASPA* gene to check for the coding pathogenic variants of patients I, II, IV, and V. The RT-PCR products were processed using the Ligation Sequencing Kit V14 (SQK-LSK114) and individually sequenced on previously used PromethION flow cells that had been nuclease treated with ONTs Flow Cell Wash Kit (EXP-WSH004).

### PCR and Sanger sequencing

REDTaq (Sigma) was used to validate the SNV c.236 + 1187A>C (NC000017.10:3380876A>C, hg19) in intron 1 of the *ASPA* gene. This PCR product was Sanger sequenced on a SeqStudio 8 Flex Genetic Analyzer (Applied Biosystems) and analyzed with Mutation Surveyor v5.1 software. PrimeSTAR^®^ HS DNA Polymerase with GC Buffer (TaKaRa) was used to amplify the SVA_E and flanking regions in intron 4 of the *ASPA* gene with a two-step PCR protocol and 100 ng DNA according to manufacturer’s instructions. To prove target specificity, these PCR products were processed using the Ligation Sequencing Kit V14 (SQK-LSK114) and individually sequenced on previously used PromethION flow cells that had been nuclease treated with ONTs Flow Cell Wash Kit (EXP-WSH004). PCR primers are available in the Supplementary section (Supplementary Table S1).

### Data analysis

#### Short-read sequencing.

The FastQC software was employed for the purpose of data quality control. The BWA-Mem algorithm was utilized to align the clean data to the reference human genome (GRCh37/hg19). The identification of candidate splice sites was conducted using SpliceAI (v1.3.1). The Genome Analysis Toolkit was used to identify single-nucleotide polymorphisms (SNPs) and copy number variants. Once identified, the variant effect predictor predicts the functional effects of the genomic variants. Coding sequence variants were classified according to the guidelines of the American College of Medical Genetics and Genomics and the Association for Molecular Pathology (ACMG/AMP criteria).^22^

#### Long-read sequencing.

The Dorado basecaller (0.7.0) was employed to convert the raw Oxford nanopore data into canonical bases of DNA or RNA. The data were aligned to the reference human genome (GRCh37/hg19) using the Minimap2 alignment program. NanopoReaTA was used for monitoring and analyzing real-time sequencing at the cDNA level.^23^ The identification of genetic variants was performed by DeepVariant (1.6) analytical pipeline. Sniffles2, a rapid structural variant (SV) caller for LRS, identified SVs. The SpliceAI algorithm predicted candidate splice sites, while the variant effect predictor assessed the functional effects of the genomic variants. Mitochondrial DNA (mtDNA) haplotyping was performed according to Behar et al. in patients I–V.^24^ Patients were assigned to mitochondrial haplogroups based on their mtDNA sequences. Hypervariable segments HVSI and HVSII were analyzed, along with specific SNPs, to determine haplogroup assignments. Haplogroup determination was performed through sequencing and comparison of HVSI and HVSII regions, commonly used for matrilineal ancestry tracing.

## RESULTS

### Clinical characteristics of CD patients

Clinical characteristics of patients I–V are reported in the Supplementary Data and summarized in Table 1. Patients I–IV exhibited a classical CD phenotype, while patient V presented with a milder form of the disease.

### ACMG/AMP-based classification of sequence variants

In all five patients, a pathogenic or likely pathogenic heterozygous variant was detected in the *ASPA* gene. Coding sequence variants previously uncovered by standard genetic diagnostics were classified by ACMG criteria and are summarized in Table 1.

### Targeted DNA nanopore sequencing identifies previously inaccessible SVs in the ASPA gene

**Figure 1.**
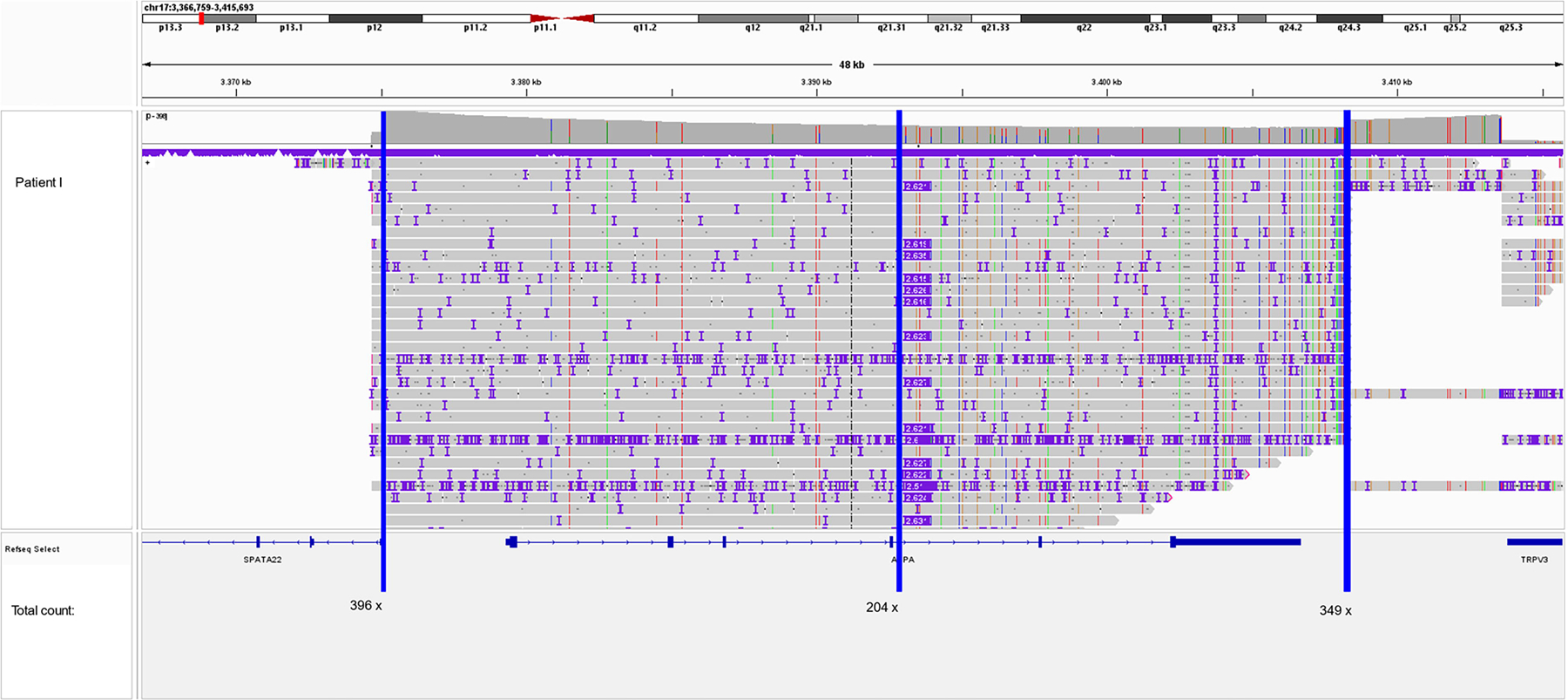
Integrative Genomics Viewer (IGV) screenshot of the *ASPA* gene of patient I. The vertical coverage of our CRISPR/Cas9 assay on an R10.4.1 PromethION flow cell (FLO-PRO114M) ranges from ~200× at the center of the horizontal coverage to 350–400× at the cut sites of the Cas9 enzyme (*blue lines* mark the reading point).

**Figure 2.**
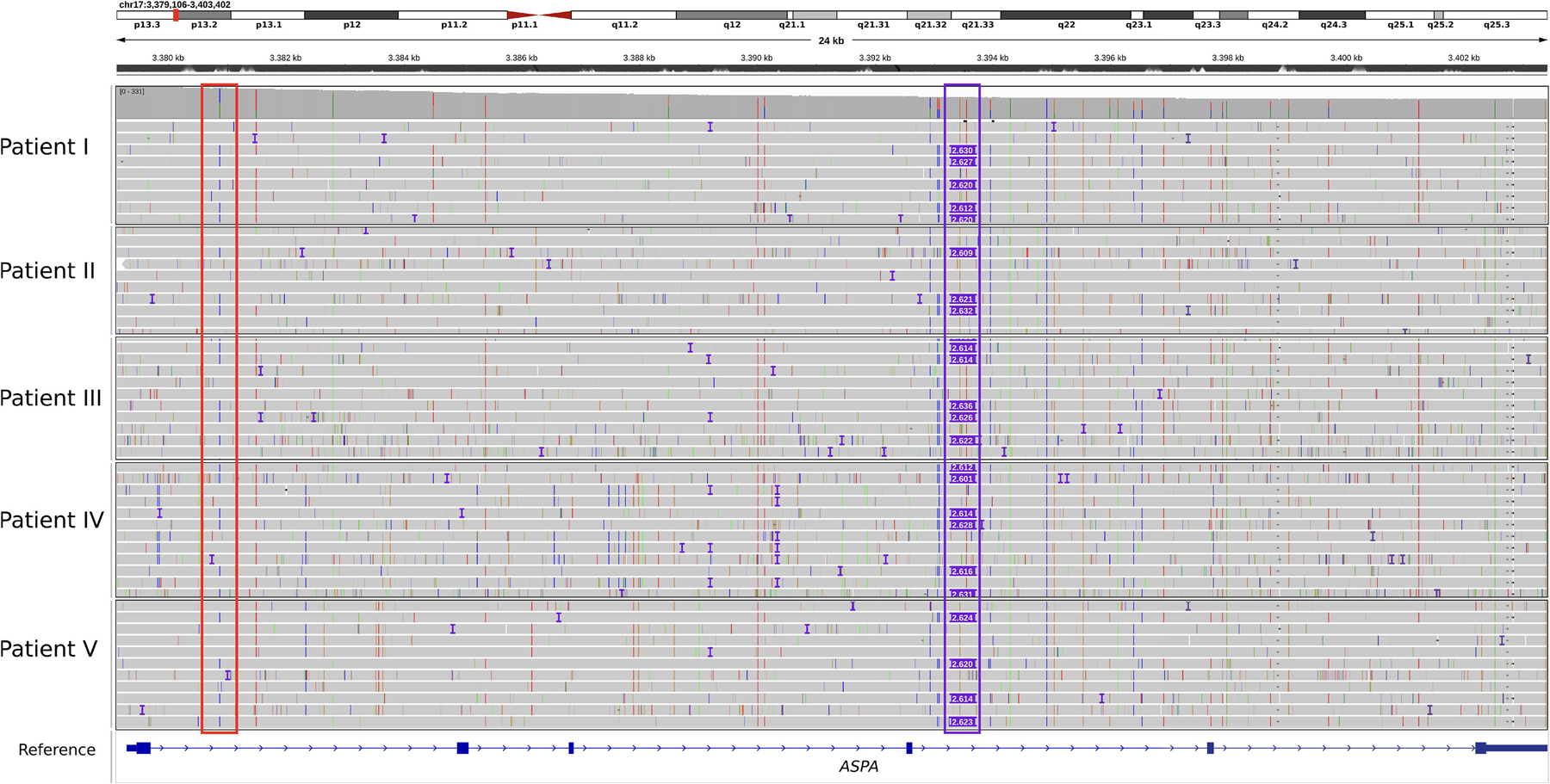
IGV screenshot of the *ASPA* gene of patients I–V. *Red outlined: ASPA* c.236 + 1187A>C in intron 1, *purple outlined*: SVA_E retrotransposon in *ASPA* intron 4. In all patients, *ASPA* c.236 + 1187A>C shares the same haplotype with the SVA_E retrotransposon.

Designed to enable a comprehensive analysis of the *ASPA* gene with high vertical coverage, our CRISPR/Cas9-based assay covers more than 38 kb encompassing the entire *ASPA* gene and flanking regions (Fig. 1). The vertical sequencing depth of all patients ranged between 100× and 300×. LRS data of all five patients revealed a heterozygous insertion with a size of ~2,630 bp in intron 4 of the *ASPA* gene (Fig. 2). LRS showed that the insertion sequence consisted of the following domains: (CCCTCT)n-like sequence, Alu-like sequence, VNTR, SINE-R, and poly(A) tail. A further refined analysis of the inserted sequence using the Dfam and RepeatMasker database revealed an SINE-VNTR-Alu (SVA) retrotransposable element of the SVA_E class and results in the following nomenclature after recommendation by the Human Genome Variation Society^25,26^:

$$\text{seq}\left[\text{GRCh}37\right]\;\text{NC}\_000017.10:\text{g}.3393511\_3393512\text{insSVA}\_\text{E}$$

**Figure 3.**
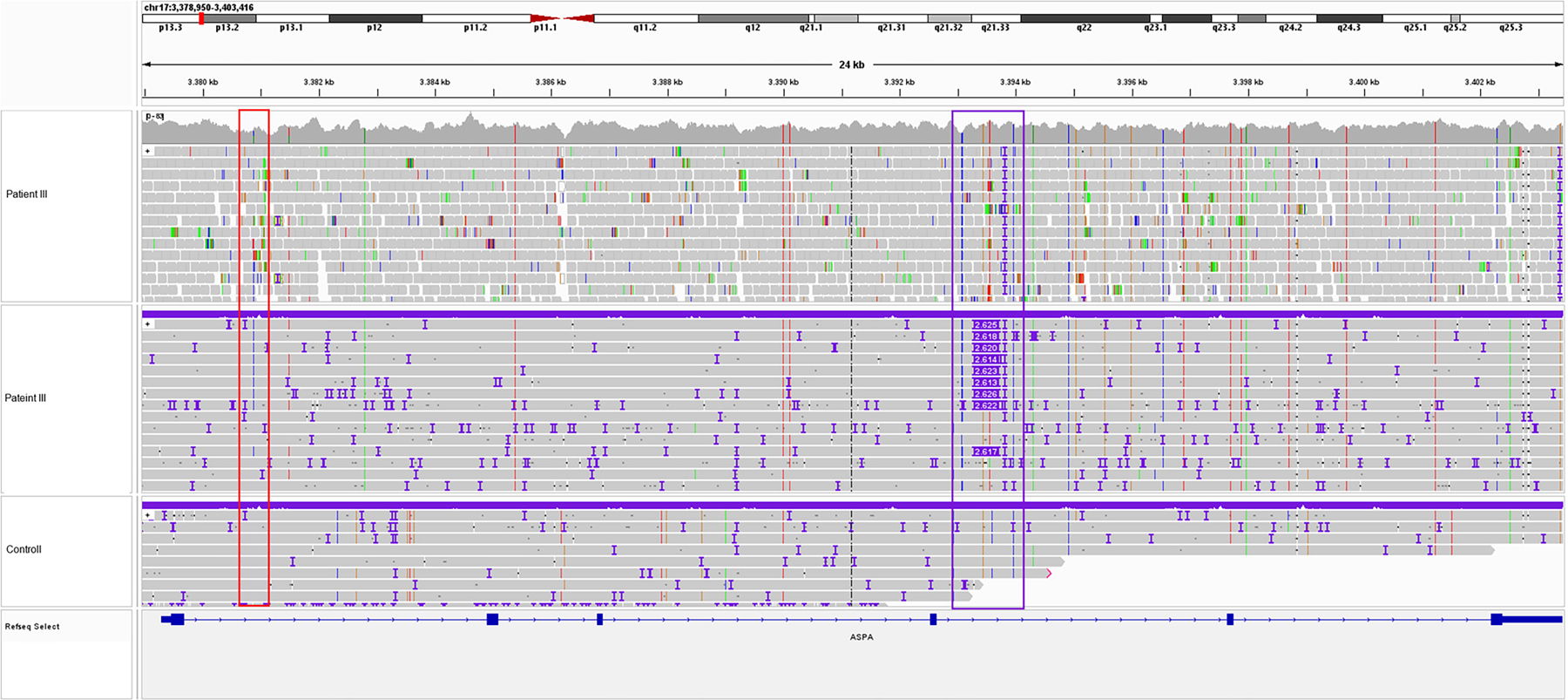
IGV screenshot of the intronic variant and an SVA_E insertion in *ASPA.* WGS data (*top*, patient III) and long-read sequencing (LRS) data (*below*, patient III and control) showing the intronic variant (*red border*) and the SVA_E insertion (*purple border*).

Linkage analysis showed a shared haplotype of the SVA_E with the variant c.236 + 1187A>C (NM_000049.4) in intron 1 which, in turn, is present in a compound heterozygous constellation with the already known pathogenic coding variants of each patient (Table 1). This result was consistent across all five patients. As an SV, this insertion was not displayed in the short-read Whole Genome Sequencing (WGS) analysis performed with standard analysis parameters (Fig. 3). A standard PCR targeting the SVA_E retrotransposon in intron 4 of *ASPA* was established, resulting in a 260 bp band (unaffected allele) and an ~3,000 bp band (allele with SVA_E insertion) for patients I–V (Supplementary Fig. S4A). Target specificity of the PCR assay was proven by LRS of the PCR products. For this purpose, an artificial reference sequence was generated from the original SVA_E-LRS data-obtained CRISPR/Cas9 and the flanking regions of intron 4 of the *ASPA* gene targeted by this PCR assay. This reference was utilized for aligning the LRS data obtained by sequencing the PCR products (Supplementary Fig. S4B).

### Colocalization of ASPA c.236 + 1187A>C and the SVA_E element on the same allele

Interestingly, the intronic variant c.236 + 1187A>C (NC_000017.10:g.3380876A>C) originally identified in patient III by short-read WGS is shared by all five patients and is located *in cis* with the SVA_E element. This variant has not been previously described in the context of CD but is listed in the Genome Aggregation Database (gnomAD) Exomes 278 times in a heterozygous state and once in a homozygous state with a variant allele frequency (VAF) of 0.00887. With a VAF of 0.0347%, the prevalence is highest among the Ashkenazi Jewish population. SpliceAI analysis predicts a gain of a splice donor site at pre-mRNA position 33 within intron 1, with a Δ score of 0.11. That score can be interpreted as a very low probability that the variant affects splicing at any position within a window of ± 500 bp around it. The variant is listed as rs191700855 in dbSNP.

### mtDNA haplotyping

Most likely based on DNA breakage during the CRISPR/Cas9 enrichment procedure and preferential sequencing of shorter DNA fragments by LRS, the mtDNA of each patient was covered well in the LRS data (mean vertical coverage: 100×). Given the high frequency of the variant *ASPA* c.236 + 1187A>C, we used these data to investigate indications of a possible founder effect based on mtDNA haplotyping. SNP-based analysis classified two patients (I, V) within haplogroup U, while the other patients (II–IV) belonged to haplogroup H (Supplementary Table S2). Haplogroup H is predominantly associated with neolithic farmers in Europe, while haplogroup U is linked to early hunter–gatherer populations. Both haplogroups are common within Ashkenazi Jewish lineages, providing a genetic marker for shared ancestry.

### Functional characterization of the SVA_E insertion

**Figure 4.**
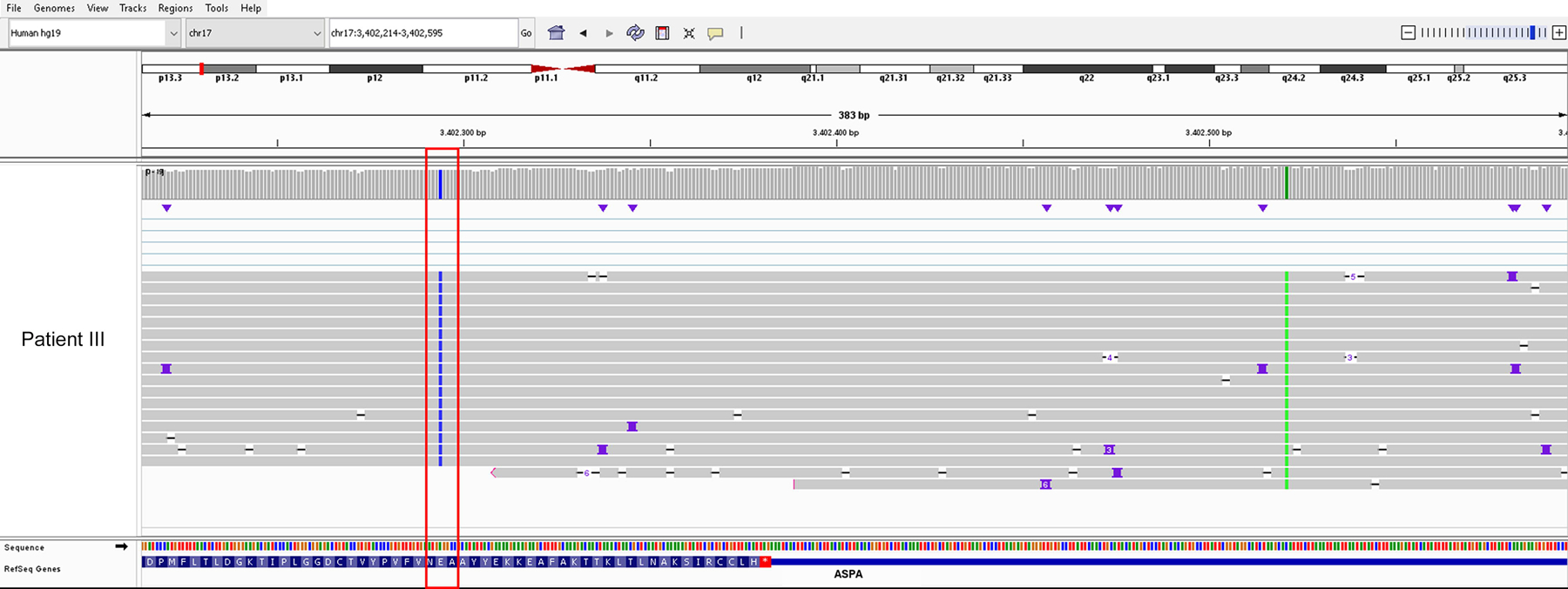
IGV screenshot of transcriptome-wide cDNA-PCR-based LRS of patient III. The pathological coding variant c.854A>C (*red-framed*) located in exon 6 is determined to be heterozygous at the DNA level but appears homozygous at the mRNA level. The polymorphism c.*139C>A in the 3′UTR of the *ASPA* gene (*green vertical line*) is homozygous on both nucleic acids.

**Figure 5.**
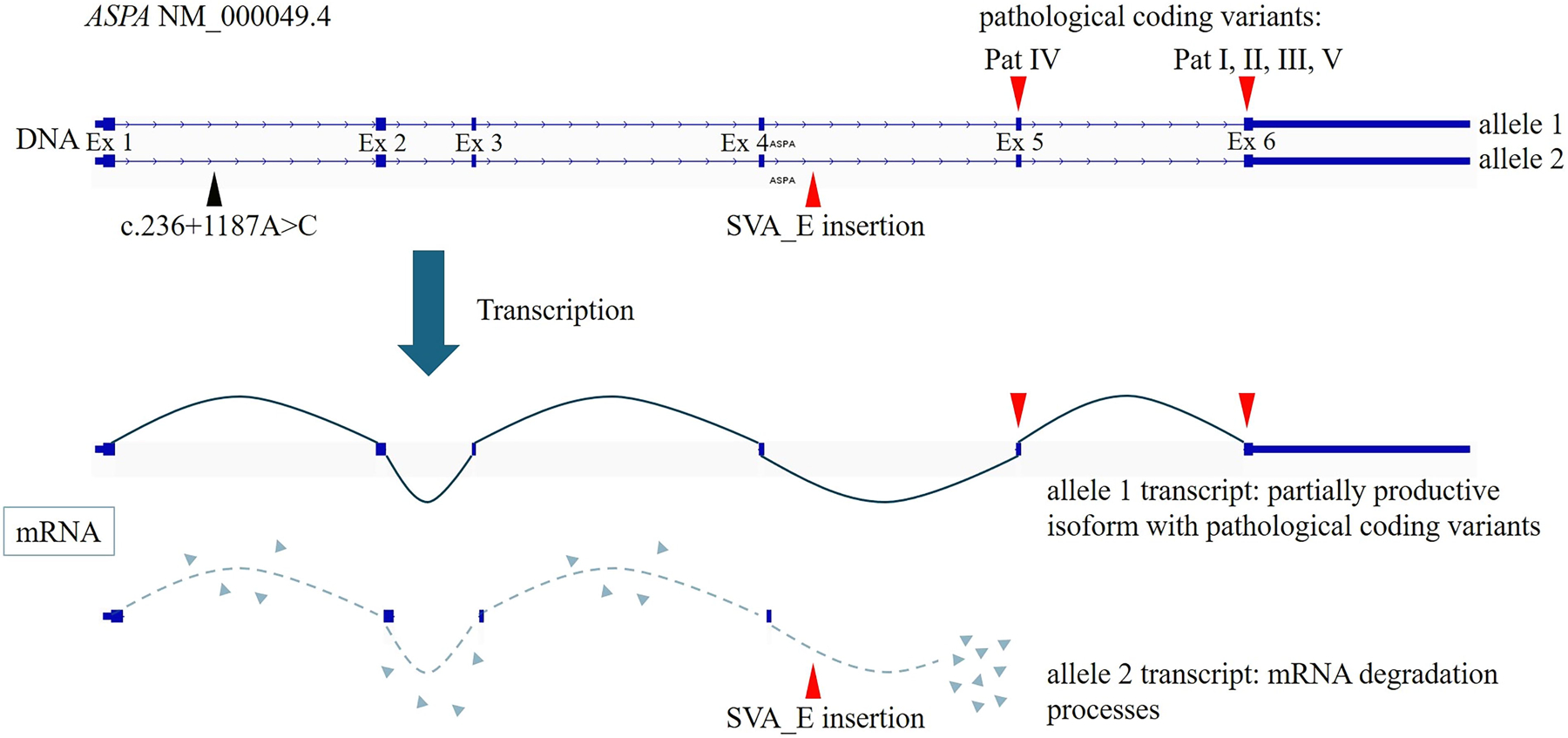
Schema of the *ASPA* gene locus at chromosome 17. Highlighted is the compound heterozygous constellation consisting of a pathogenic coding variant from the respective patients (allele 1) and the SVA_E retrotransposon (allele 2). During transcription, this results in a partially productive isoform (allele 1 transcript), which is determined by the severity of the respective coding variant, and an isoform that undergoes mRNA degradation (allele 2 transcript) and is no longer detectable via LRS-based transcriptome-wide cDNA sequencing.

To gain insights of possible damaging effects of the SVA_E element on *ASPA* transcripts, we performed transcriptome-wide cDNA-PCR-based LRS in sample III. The patient showed homozygosity for the pathogenic coding variant that has been found heterozygous on the DNA level (Fig. 4). With this comprehensive and unbiased analysis, no evidence of potentially expressed truncated isoforms caused by the SVA_E element could be observed. RT-PCR followed by LRS analysis of patients I, II, IV, and V confirmed homozygosity for their respective coding pathogenic variants as well (Supplementary Figs. S2 and Figs. S3). Overall, these data suggest the loss of the SVA_E-associated transcripts (Fig. 5).

## DISCUSSION

This study expands the understanding of the molecular causes of CD by identifying a heterozygous SVA_E retrotransposon insertion in intron 4 of the *ASPA* gene. The length of SVAs varies from a few hundred to several thousand base pairs, depending on the size of their hexamer and VNTR regions. These elements are evolutionarily young and exclusive to hominids. They are classified based on their age SVA_A to SVA_F, with SVA_E and SVA_F being restricted to the human lineage.^27,28^ Approaches based on short-read sequencing for detecting SVAs are limited by their repetitive sequences and highly GC-rich regions, indicating that the pathogenic contribution of SVAs is significantly underdiagnosed. Our CRISPR/Cas9-based approach for targeted enrichment of the entire *ASPA* gene achieved an average vertical minimum coverage of over 100× per patient on a single PromethION flow cell. From a scientific perspective, this approach allows for a precise analysis of subtle DNA methylation changes induced by retrotransposition. Additionally, from an economic perspective, the approach is also suitable for multiplexing, enabling the analysis of multiple patients simultaneously. Given that LRS is not universally accessible in molecular genetics laboratories, we have established a PCR-based assay that enables the detection of the SVA_E element in intron 4 of the *ASPA* gene, utilizing standard laboratory equipment. The target specificity of the PCR assay was confirmed using LRS.

Further linkage analysis showed a shared haplotype with a conserved intronic variant (*ASPA* c.236 + 1187A>C) in all five patients analyzed in this study. This haplotype showed a compound heterozygous constellation with known pathogenic coding variants of each patient. Functional characterization of this SV at the mRNA level revealed homozygosity for the specific coding variants of each individual patient, which must be accompanied by a loss of the transcripts altered by the SVA_E retrotransposition. Our data highlight the critical importance of functional studies on inserted elements in noncoding regions and their potential role in disease pathogenesis. mtDNA haplotyping confirms that all five patients evaluated in this study are of Ashkenazi Jewish descent, a population characterized by a higher prevalence of certain genetic disorders. All patients showed one haplotype consisting of the intronic variant c.236 + 1187A>C *in cis* with the SVA_E and *in trans* to their specific pathogenic exonic variant.

It is crucial to highlight that a complete understanding of the genetic causes of CD is necessary for patients to participate in gene therapy trials that are currently ongoing (NCT04833907, NCT04998396) and for further family planning. In CD patients, variants of uncertain significance or the absence of a second heterozygous variant, despite a clear phenotype, pose significant challenges for evaluating ASPA residual activity and potential gene therapy-related cytotoxicity by potential overexpression. The clinical examination of our five patients revealed four affected individuals with a typical CD phenotype and one affected individual with a mild phenotype. While variants found in patients I–III were all associated with severe phenotypes,^4,29,30^ variant c.863A>G (patient V) was previously reported to be associated with a milder phenotype of CD.^31,32^ The c.740T>C variant was previously reported, but without a phenotype association.^33^

Since all of our patients share the same haplotype of SVA_E and *ASPA* c.236 + 1187A>C, it seems that only the exonic variant correlates with the severity of the course of the disease, while the SVA_E-associated allele has a more supporting role. The SVA_E insertion likely generates a new splice acceptor site within intron 4 of *ASPA*, leading to a disruption of the regular splicing process. These findings facilitate patients’ access to current gene therapy trials as heterozygous patients are often not considered as candidates for interventions, due to the increased risk of overexpression of the ASPA enzyme and the associated toxicity. Additionally, these findings open up an exciting opportunity for the development of a splice-switching antisense oligonucleotides CD therapy in patients who are appropriately affected. While identifying individuals with genetic variants amenable to such approaches remains challenging,^34,35^ Kim et al. introduce a framework designed to systematically identify and develop splice-switching therapies for rare diseases.^36^ Within this framework, deep intronic SVs that interfere with canonical splicing are considered particularly suitable targets for this therapeutic approach. The feasibility of such a treatment strategy was also demonstrated by Yano et al. for the occipital horn syndrome, the molecular cause of which is a deep intronic insertion of an SVA_D element.^14^

In conclusion, we have been able to confirm previously molecularly unresolved cases of CD. The underlying cause is the insertion of an SVA_E retrotransposon into intron 4 of the *ASPA* gene. For affected patients and their families, these findings enable precise genetic counseling, particularly regarding reproductive planning. Furthermore, these findings facilitate patients’ access to current gene therapy trials, as heterozygous patients are often not considered candidates for interventions, due to associated risks. Experimentally confirmed, the SVA_E insertion leads to highly efficient mRNA degradation processes and thus offers potential innovative therapeutic approaches in the context of splice-switching antisense oligonucleotides. The identification of an intronic variant may be exemplary for other undiagnosed monogenetic diseases and may open doors for potentially new gene therapy approaches.

## Supplementary Material

Supplemental Data

Supplemental figure 1

Supplemental figure 2

Supplemental table 1

Supplemental figure 3

Supplemental figure 4

Supplemental table 2

Supplementary Data

Supplementary Figure S1

Supplementary Figure S2

Supplementary Figure S3

Supplementary Figure S4

Supplementary Table S1

Supplementary Table S2

## Figures and Tables

**Table 1.** Summary of Clinical Characteristics in Five Canavan Disease Patients

*Individual*	*I*	*II*	*III*	*IV*	*V*
Exonic variant	c.878_880delAAG	c.914C>A	c.854A>C	c.740T>C	c.863A>G
	p.Glu293del	p.Ala305Glu	p.Glu285Ala	p.Leu247Pro	p.Tyr288Cys
ACMG classification (exonic variant)	PS3+PM2+PM4+PM1+PP5	PM3+PS3+PM2+PM1+PP3	PM3+PP1+PS3+PP3+PM2+PP2	PP3+PM2+PP2_	PP3+PM2+PM5+PP2
	4 + 1 + 2 + 2 + 4 = 13	8 + 1 + 1 + 1 + 1 + 1 = 13	8 + 1 + 2 + 4 + 1 + 1 = 17	4 + 1 + 1 = 6	4 + 1 + 2 + 1 = 8
	Pathogenic	Pathogenic	Pathogenic	Likely pathogenic	Likely pathogenic
Allele frequency (exonic variant)	N/A	(VAF) 0.00016	(VAF) 0.00043	N/A	N/A
		(ASJ) 0	(ASJ) 0.00922 (Founder variant)		
Intronic variant	c.236 + 1187A>C +	c.236 + 1187A>C +	c.236 + 1187A>C +	c.236 + 1187A>C +	c.236 + 1187A>C +
	SVA_E	SVA_E	SVA_E	SVA_E	SVA_E
Allele frequency (intronic variant)	(VAF) of 0.00887	(VAF) of 0.00887	(VAF) of 0.00887	(VAF) of 0.00887	(VAF) of 0.00887
	(ASJ) of 0.0347	(ASJ) of 0.0347	(ASJ) of 0.0347	(ASJ) of 0.0347	(ASJ) of 0.0347
Sex	♀	♂	♂	♀	♂
Symptoms at onset [m]	[4]^a,b,c,d,e,f^	[3]^a,g,b,h^	[3]^a,g^	[3]^a,b,h,d^	[7]^a,c^
Age at diagnosis [m]	7	4	7	4	21
HC at last follow-up [y]	2.88 z, >99th P. [10]	8.56 z, >99th P. [5]	5.08 z, >99th P. [6]	3.43 z, >99th P. [4]	1.85 z, 97th P. [6]
Walking with support	–	–	–	–	+
Speaking words	–	–	–	–	+
Tube feeding	+	+	–	–	–
Seizures	+	–	+	–	–
Max. total CDRS [y]	15 [9]	19 [5]	21 [5]	16 [5]	5 [5]
Min. total CDRS [y]	14 [7]	16 [2]	16 [3]	16 [4]	3 [6]
Phenotype	Typical	Typical	Typical	Typical	Mild

ACMG variant classification is variant classification according to the Recommendations of the American College of Medical Genetics and Genomics.^20^

Canavan disease rating score (CDRS) severity 0–22 points.^4^

a Developmental delay.

b Reduced head control.

c Muscular hypotonia.

d Abnormal eye movements.

e Poor vision.

f Irritability/startling.

g Macrocephaly.

h Muscular hypertonia.

(+), present; (−), absent; ASJ, allele frequency across Ashkenazi Jewish population; HC, head circumference; [m], age in months; N/A, not assessed; P., percentile; SVA_E, SVA element; VAF, variant allele frequency; [y], age in years.
