# Supplementary material for: Deep Intronic SVA_E Retrotransposition as a Novel Factor in Canavan Disease Pathogenesis
Source: Hum Gene Ther. 2025 Sep 12;36(17-18):1248–56. doi: 10.1089/hum.2025.006 (PMC12596875; doi:10.1089/hum.2025.006)
Supplement: Supplementary file 1 [file hum.2025.006_supplementary_data.pdf]

Supplement: Case reports

*Patient I***:** The female patient was the result of an uncomplicated 39-week pregnancy of non-consanguineous parents. Her postnatal course was unremarkable. First symptoms appeared at the age of 4 months and included poor head control, muscular hypotonia, macrocephaly, strabismus, and irritability. Visual tracking was reduced. The diagnosis of Canavan disease was made at the age of 7 months by pathologic elevation of NAA in MRS and urine. She acquired the ability to roll over at 6 months but lost this ability at 11 months. She learned grasping and hand-to-hand transfer at 6 and 7 months, respectively, but lost these skills by 14 months. Head control, toilet training, unsupported sitting, and walking were never achieved. From the age of 7 years, the spasticity continued to increase, and gaze fixation decreased. A gastric tube was placed at the age of 9 years. The patient was able to communicate with her family using a communication device. At 10 years and 2 months, she experienced her first seizure episode.

Patient II: The male patient was born to non-consanguineous parents at 40+5 weeks' gestational age. Apart from a neonatal infection, the postnatal course was unremarkable. The diagnosis of Canavan disease was made at 3 months of age with proof of pathologically elevated NAA in urine and MRS. The first symptoms included poor head control, lack of gaze fixation, macrocephaly, limb spasticity, and irritability to noise and light. A gastric tube was placed at 16 months of age. The first seizure occurred at 25 months. At 28 months, the patient was able to babble and sit with support. He never developed the ability to speak words or sentences, grasp objects, to sit without support nor to walk.

Patient III: The male patient was the result of a 40-week pregnancy of non-consanguineous parents. The postnatal course was unremarkable. The patient gained limited head control and was able to support himself in the prone position but lost these abilities by the age of 9 months. He exhibited truncal hypotonia, limb spasticity, and macrocephaly. Diagnosis was confirmed by pathologic NAA-levels in urine. Starting at 21 months, frequent seizures were reported. The ability to track visually was observed at 30 months but was lost by the age of 3 years and 10 months. At this age, he also lost the ability to vocalize and laugh aloud, showing fewer emotional reactions. Spasticity and swallowing problems increased. Sufficient head control, grasping, independent sitting, and walking were never achieved.

Patient IV: The female patient was born at 37 weeks of gestation by caesarean section. After mild asphyxia and hyperbilirubinemia, the postnatal course was unremarkable. The parents are non-consanguineous. By 2 months of age, poor head control, abnormal visual fixation, delayed motor development, and limb spasticity were noticed. NAA-levels in urine were pathologically elevated. Macrocephaly was reported by 4 years of age. At 5 years old, the patient was babbling and showed smiling and crying. Speaking, grasping, unsupported sitting, or standing were never learned. No seizures have been reported.

Patient V: The male patient was born at 39 weeks' gestation to non-consanguineous parents via caesarean section. Gaze fixation was achieved within a few weeks. At 4-5 months, the patient was able to grasp objects. By 6 months, developmental delay and muscular hypotonia of the lower limbs were noted. Diagnosis of Canavan Disease was confirmed by pathologically elevated NAA-levels in urine. He was able to turn at 10 months, to sit without support at 13 months, and to crawl at 18 months. The patient was able to walk with support at 2 years and 6 months, and walk without support at 3 years and 3 months. However, by the age of 5 years, walking was only possible with support. The patient can speak single words and communicates using sounds and gestures. He has developed basic fine motor skills and is toilet trained. Macrocephaly was not observed.
