# Supplementary material for: Deep Intronic SVA_E Retrotransposition as a Novel Factor in Canavan Disease Pathogenesis
Source: Hum Gene Ther. 2025 Sep 12;36(17-18):1248–56. doi: 10.1089/hum.2025.006 (PMC12596875; doi:10.1089/hum.2025.006)
Supplement: Supplementary file 3 [file hum.2025.006_supplementary_figures2.pdf]

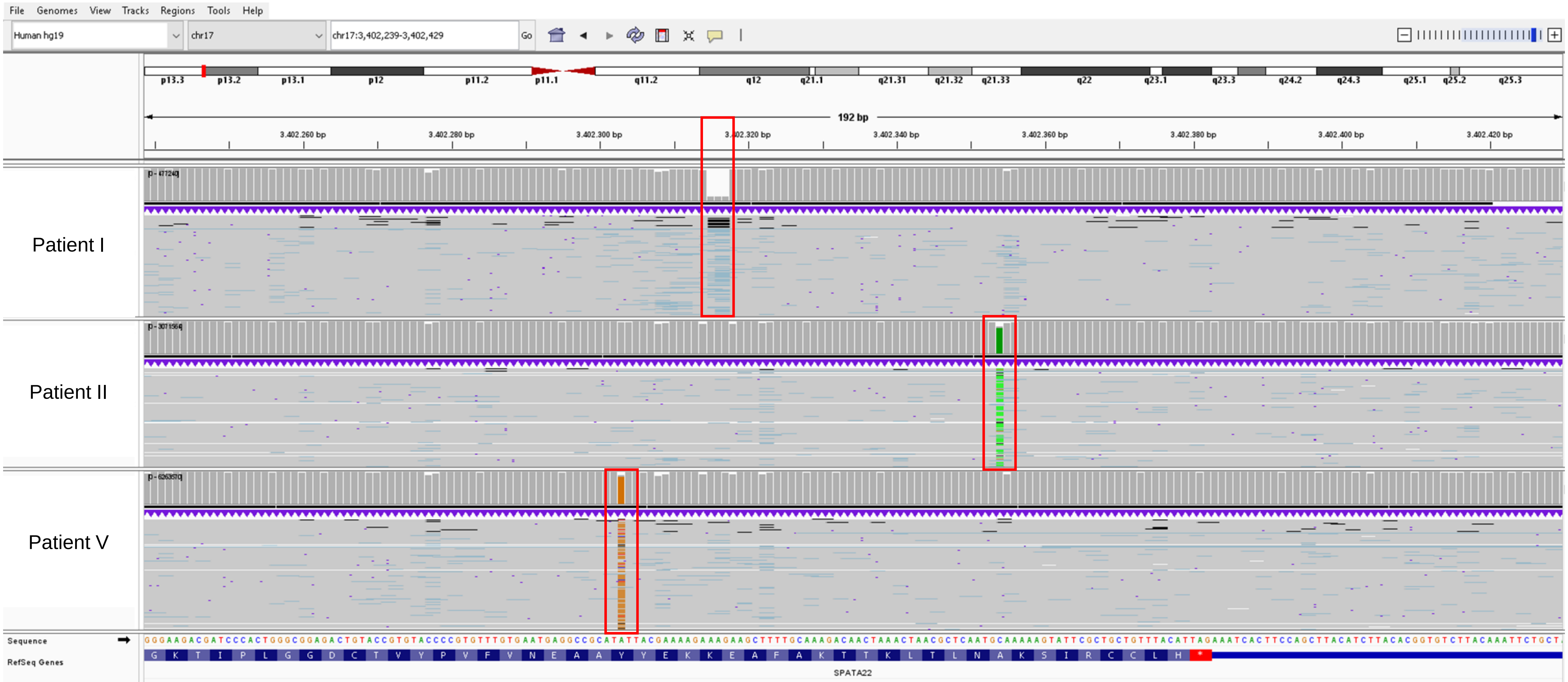

Suppl. Fig. 2: RT-PCR followed by LRS showing homozygosity for the specific coding variants of patient I, II and V in exon 6 of the *ASPA* gene (Red-framed). Patient I: c.878_880delAAG; patient II: c.914C>A; patient V: c.863A>G
