# Supplementary material for: Deep Intronic SVA_E Retrotransposition as a Novel Factor in Canavan Disease Pathogenesis
Source: Hum Gene Ther. 2025 Sep 12;36(17-18):1248–56. doi: 10.1089/hum.2025.006 (PMC12596875; doi:10.1089/hum.2025.006)
Supplement: Supplementary file 4 [file hum.2025.006_supplementary_figures3.pdf]

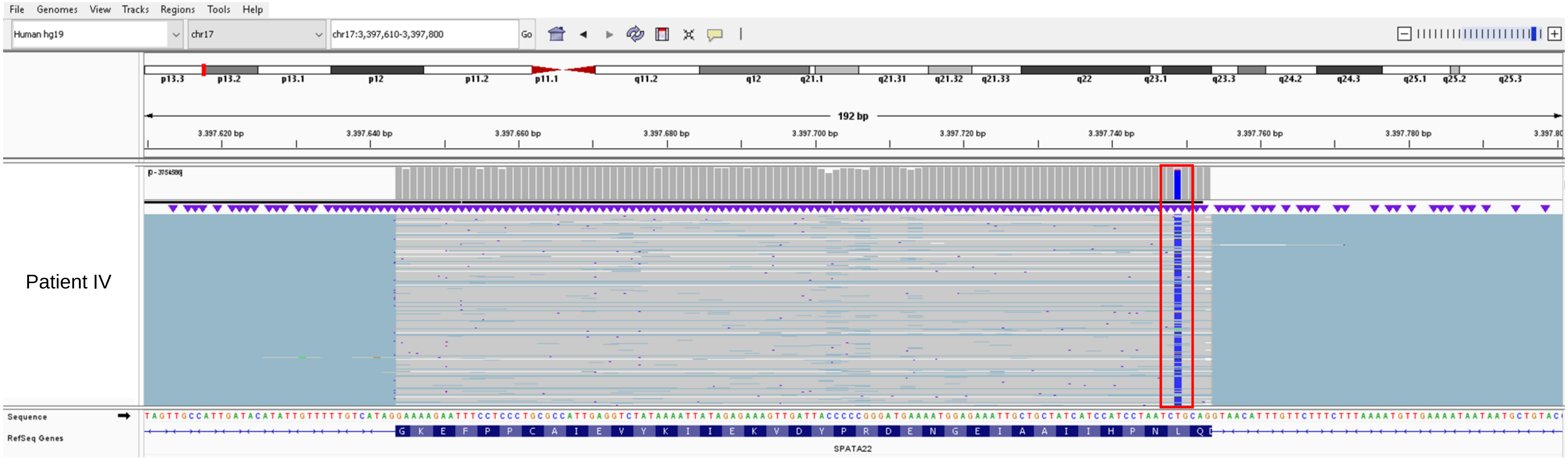

Suppl. Fig. 3: RT-PCR followed by LRS showing homozygosity for the specific coding variant of patient IV in exon 5 of the *ASPA* gene (c.740T>C, Red-framed).
