# Supplementary material for: Deep Intronic SVA_E Retrotransposition as a Novel Factor in Canavan Disease Pathogenesis
Source: Hum Gene Ther. 2025 Sep 12;36(17-18):1248–56. doi: 10.1089/hum.2025.006 (PMC12596875; doi:10.1089/hum.2025.006)
Supplement: Supplementary file 5 [file hum.2025.006_supplementary_figures4.pdf]

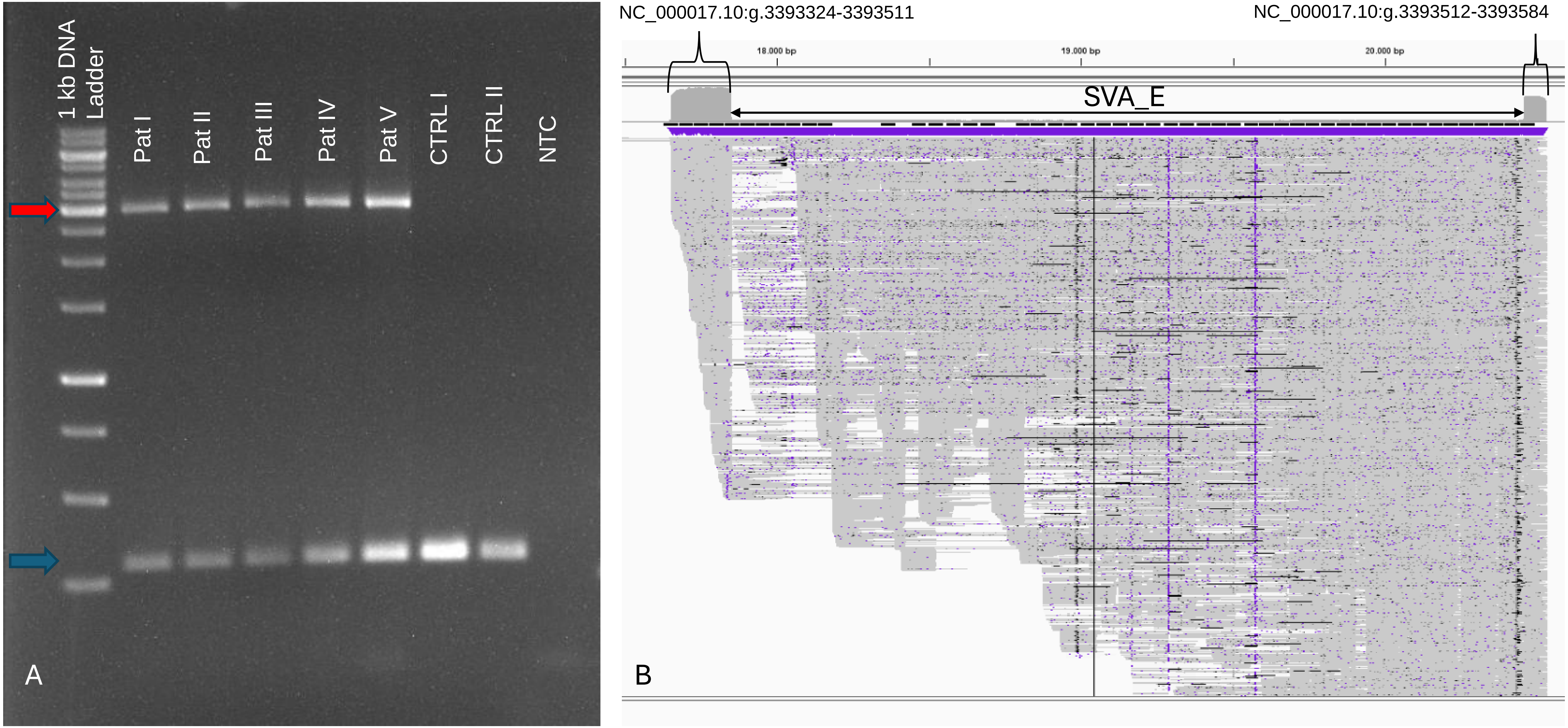

Suppl. Fig. 4: PCR flanking the SVA_E in intron 4 of *ASPA*. A: Patients I-V show 2 PCR bands for unaffected (260 bp, blue arrow) and affected allele (≈ 3 kb, red arrow). B: IGV screenshot confirming the PCR target specificity by LRS. The flanking regions of *ASPA* intron 4 marked with brackets and annotated with genomic coordinates (GRCh37/hg19) show significantly higher coverage due to preferential LRS of smaller fragments presented by the 260 bp wildtype allele.
