# Supplementary material for: Deep Intronic SVA_E Retrotransposition as a Novel Factor in Canavan Disease Pathogenesis
Source: Hum Gene Ther. 2025 Sep 12;36(17-18):1248–56. doi: 10.1089/hum.2025.006 (PMC12596875; doi:10.1089/hum.2025.006)
Supplement: Supplementary file 6 [file hum.2025.006_supplementary_tables1.pdf]

Suppl. Tab. 1: CRISPR/Cas9 crRNA design, PCR primer sequences *ASPA* c.236+1187A>C, exon-bridging RT-PCR primer (Exon 5 and 6) and SVA_E flanking PCR primers

Category	***Target Sequence / 5'-3' Sequence***	***Genomic Location (GRCh37/hg19)***	***Strand***	***Annealing Temperature [° C]***	***Product Length [bp]***
**crRNA**	TCTCAAAGCGCATTCACATCAGG	chr17:3510297	-		
**crRNA**	TCTTATGTGGCCAAATTGCTGGG	chr17:3505025	-		
**crRNA**	GCGAAGAAAGCGCGGGAATCAGG	chr17:3471800	+		
**crRNA**	TAAGTGTAACATTCCACCCGCGG	chr17:3471373	+		
**SNV Intron 1 PCR For**	GCCTTCGTTTACATCATAATAGCTACC	chr17:3,380,674-3,380,700 (*ASPA* Intron 1)		59.0	239
**SNV Intron 1 PCR Rev**	GTAGAAACCAAGTTGTATAGAGCTG	chr17:3,381,126-3,381,150 (*ASPA* Intron 1)			
**RT-PCR For**	AGCCAAGTATCCTGTGGGTAT	Exon bridging 689/690 (forward primer) on template NM_000049.4		60.0	550
**RT-PCR Rev**	AGGCACTGTGCTAGGAGCTA				
**Flanking PCR For**	TGCAGACCAAGACATTCAGTACA	chr17:3,393,324-3,393,346 (*ASPA* Intron 4)		2-step protocol accord. to manufacturer	260 bp unaffacted ≈ 3000 bp affected
**Flanking PCR Rev**	TACCCACCTTGTCCACCAGT	chr17:3,393,565-3,393,584 (*ASPA* Intron 4)			
