# Supplementary material for: Deep Intronic SVA_E Retrotransposition as a Novel Factor in Canavan Disease Pathogenesis
Source: Hum Gene Ther. 2025 Sep 12;36(17-18):1248–56. doi: 10.1089/hum.2025.006 (PMC12596875; doi:10.1089/hum.2025.006)
Supplement: Supplementary file 7 [file hum.2025.006_supplementary_tables2.pdf]

Suppl. Tab. 2: Assignment of patients I-V to one of the mtDNA haplogroups of the Ashkenazi Jewish population.[24]

Patient	***HVSI***	***Middle***	***HVSII***	***Hg***
I	*c.16192C>T; c.16256C>T; c.16270C>T;*	*c.16526G>A*	*c.73A>G; c.263A>G;*	***U5a1*** *Poland/Romania/ Ukraine*
II		*c.16519G>C*	*c.263A>G;*	***H*** *largest group very mixed**
III	*c.16189T>C; c.16356T>C;*	*c.16519G>C*	*c.152T>C; c.263A>G*	***H***
IV		*c.16519G>C*	*c.263A>G*	***H***
V	*c.16189T>C; c.16270C>T*		*c.73A>G; c.263A>G; c.150C>T;*	*U5b/Byelorussia*

*Latvia, Netherlands, Lithuania, Romania, Poland, Austira, Hungary, Russia, Germany
