# Supplementary material for: Deep Intronic SVA_E Retrotransposition as a Novel Factor in Canavan Disease Pathogenesis
Source: Hum Gene Ther. Author manuscript; Available in PMC 2025 Nov 9. (PMC12596875; doi:10.1089/hum.2025.006)
Supplement: Supplemental figure 1 [file NIHMS2119170-supplement-Supplemental_figure_1.pdf]

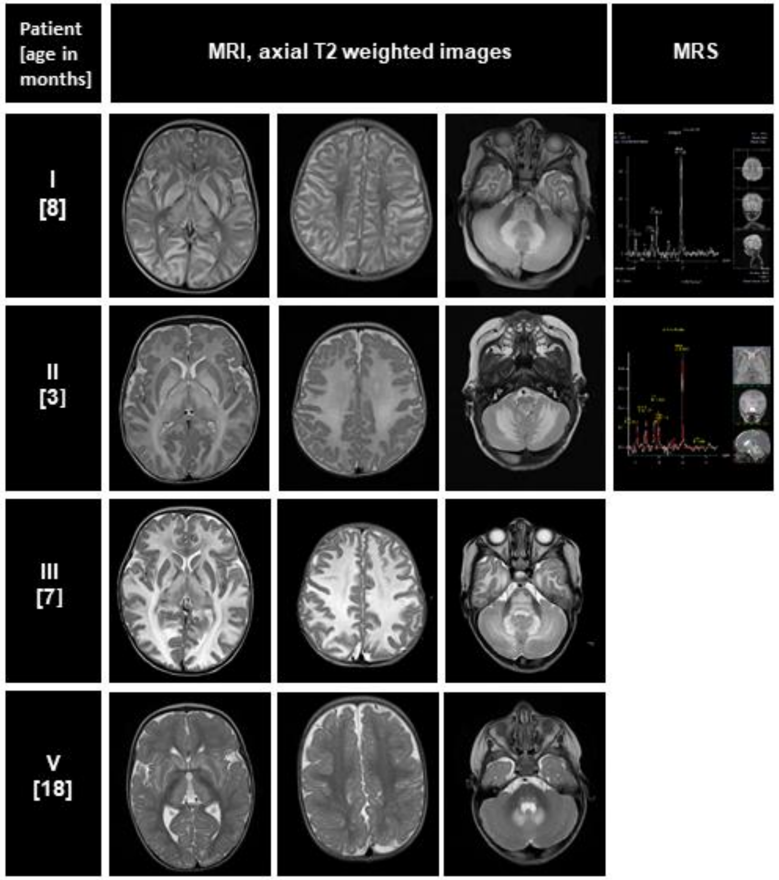

Suppl. Fig. 1: Axial T2-weighted brain MR images (MRI) of patients I, II, III and V and MR spectroscopy (MRS) of patient I and II. MRI findings in patient I, II, and III reveal symmetrical signal hyperintensities in the supra- and infratentorial white matter, globus pallidus, thalamus, brainstem, and cerebellum, indicative of extensive hypomyelination. The putamen and caudate nucleus are spared, consistent with the characteristic imaging pattern of Canavan disease. The MRI of Patient V shows less pronounced signal hyperintensities in the subcortical white matter, cerebellum and brainstem as well as bilateral affection of the putamen, caput caudate nucleus, and thalamus, with sparing of the globus pallidus. MRI of patient V is suggesting a milder phenotype of CD. MRS in patients I and II demonstrates an abnormal N-acetylaspartate peak, consistent with Canavan disease.[7]
