# Supplementary material for: Deep Intronic SVA_E Retrotransposition as a Novel Factor in Canavan Disease Pathogenesis
Source: Hum Gene Ther. Author manuscript; Available in PMC 2025 Nov 9. (PMC12596875; doi:10.1089/hum.2025.006)
Supplement: Supplemental figure 2 [file NIHMS2119170-supplement-Supplemental_figure_2.pdf]

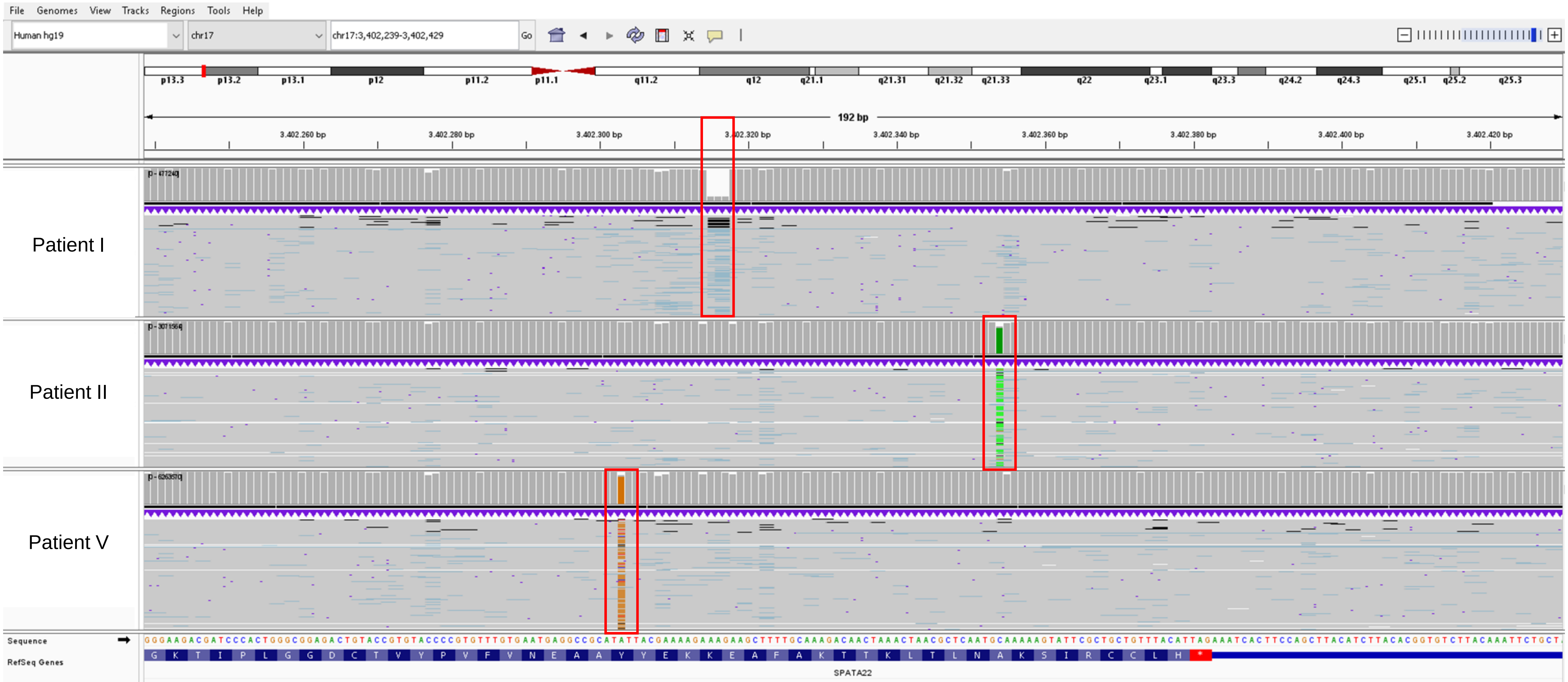

Suppl. Fig. 2: RT-PCR followed by LRS showing homozygosity for the specific coding variants of patient I, II and V in exon 6 of the *ASPA* gene (Red-framed). Patient I: c.878_880delAAG; patient II: c.914C>A; patient V: c.863A>G
