# Supplementary material for: Deep Intronic SVA_E Retrotransposition as a Novel Factor in Canavan Disease Pathogenesis
Source: Hum Gene Ther. Author manuscript; Available in PMC 2025 Nov 9. (PMC12596875; doi:10.1089/hum.2025.006)
Supplement: Supplemental figure 3 [file NIHMS2119170-supplement-Supplemental_figure_3.pdf]

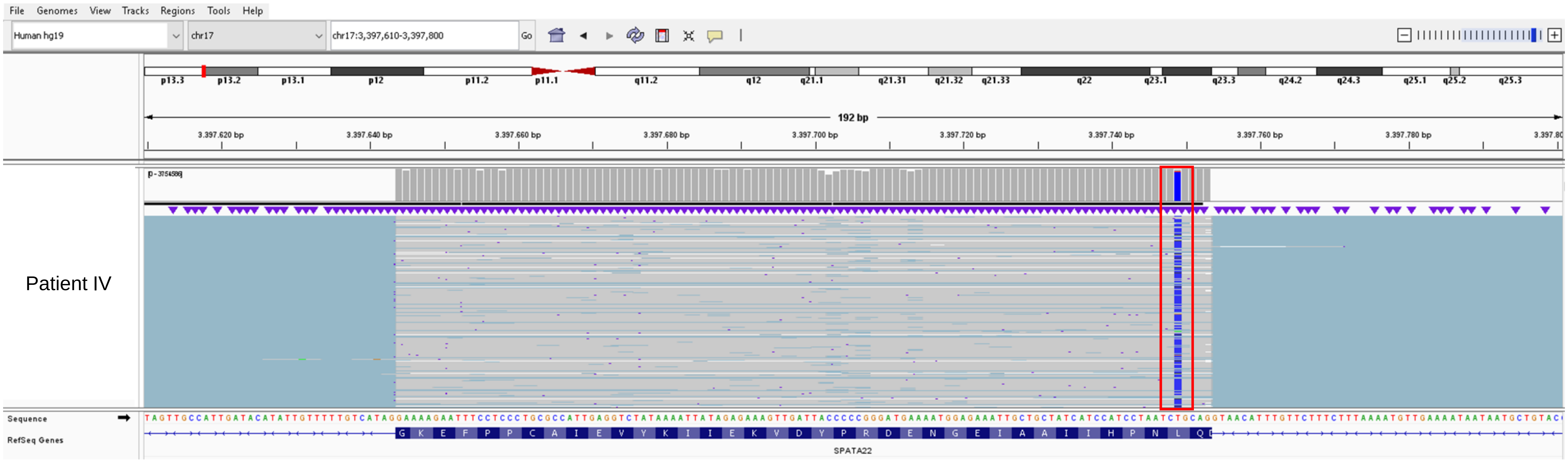

Suppl. Fig. 3: RT-PCR followed by LRS showing homozygosity for the specific coding variant of patient IV in exon 5 of the *ASPA* gene (c.740T>C, Red-framed).
